# Old Tools in a New Era: The Continued Relevance of Chemotherapy in Pediatric Neuro-Oncology

**DOI:** 10.3390/curroncol32070410

**Published:** 2025-07-20

**Authors:** Kathleen Felton, Lucie Lafay-Cousin, Sylvia Cheng

**Affiliations:** 1 Jim Pattison Children’s Hospital, Saskatoon, SK S7N 0W8, Canada; kathleen.felton@saskhealthauthority.ca; 2 Alberta Children’s Hospital, Calgary, AB T3B 6A8, Canada; lucie.lafay-cousin@ahs.ca; 3 British Columbia Children’s Hospital, 4480 Oak Street, B315, Vancouver, BC V6H 3V4, Canada

**Keywords:** chemotherapy, pediatric brain tumors, high-dose chemotherapy

## Abstract

Conventional chemotherapy continues to form the backbone of treatment for many pediatric central nervous system (CNS) tumors. Advances have been made especially in the molecular underpinning of certain pediatric CNS tumors, allowing for advancement and consideration in incorporating this molecular information in molecular targeted therapy or appropriate de-escalation or escalation of therapy. In very young children with embryonal CNS tumors, intensive high-dose chemotherapy approaches have been used with varied increased survival in medulloblastoma, atypical teratoid rhabdoid tumor (ATRT), and rare embryonal subtypes, but there are certain molecular risk groups that require new therapies, such as the ATRT MYC subtype. Some CNS tumors remain resistant or refractory to conventional chemotherapy, especially in relapsed disease. Strategies to explore combination therapies with chemotherapy, novel agents, and novel approaches are needed to improve survival in this population in the future.

## Simple Summary

Researchers are excited about new treatment possibilities for children with brain tumors, including non-chemotherapy options like targeted drugs and immune-based therapies. These may help improve survival or reduce long-term side effects. However, chemotherapy is still the main treatment for most pediatric brain tumors. For certain tumor types, especially those that respond well to chemotherapy, doctors are trying stronger combinations to improve outcomes. In other cases, like some aggressive tumors that don’t respond well to chemotherapy, researchers are searching for better alternatives. Ongoing studies are comparing standard chemotherapy with newer treatments to find the best approach. For now, chemotherapy remains a central part of treatment, but growing knowledge about each tumor type may help doctors choose more personalized therapies. Future advances may also include new drugs and better ways to deliver treatments, giving hope for better results and fewer side effects for children with brain tumors.

## 1. Introduction

Over the past few decades, advances have been made in the treatment of pediatric central nervous system (CNS) tumors, resulting in improved survival and cure rates for children. Conventional chemotherapy continues to form the backbone of many treatment regimens, but the incorporation of molecular targeted therapy, advances in apheresis, and improved supportive care measures has allowed for different ways to use chemotherapy to a child’s benefit for cure and potential reduction in late side effects.

In this review, we aim to provide an overview of pediatric brain tumor types that continue to rely heavily on chemotherapy, including high-dose regimens, despite significant molecular and biological advancements in the field. We discuss how emerging insights into tumor biology have reshaped the use of chemotherapy, refining indications, influencing treatment intensity, and informing the integration of adjunctive therapies such as radiotherapy. Finally, we revisit tumor entities that remain refractory to current treatments, where meaningful therapeutic breakthroughs have been limited, and chemotherapy remains largely ineffective.

## 2. Use of Chemotherapy in Pediatric Low-Grade Glioma

Pediatric low-grade glioma (PLGG) is the most common brain tumor in the pediatric population, comprising 30–50% of all newly diagnosed brain tumors [1], and naturally becomes the bread-and-butter of an oncologist’s clinical practice. Strategies in the treatment of PLGG have varied greatly, from “watch and wait” to multimodal therapy, including surgery, chemotherapy, radiotherapy, and/or targeted therapy. Treatment choices depend on clinical presentation, tumor location, operability, histology, and molecular profile. Chemotherapy was initially used in the 1970s as salvage therapy after failure of radiotherapy (RT). However, many international centers have endeavored to avoid RT due to side effects, despite higher PFS [2]. Current recommendations use chemotherapy as the main treatment modality for incompletely resected or inoperable tumors. Multiple studies and prospective clinical trials have been conducted with different chemotherapies, either in combination or as monotherapy, in PLGG at initial diagnosis or after progression. Overall response rates of up to 50–60% and 5-year PFS of 39–53% have been reported in most series. This has led to clinical equipoise between chemotherapy regimens [3].

The regimens combining carboplatin and vincristine (CV) have been established as one of the international standard therapies for PLGG, due to the possibility of outpatient-based administration, favorable acute (reversible) toxicity profile, and low risk of late sequelae. The carboplatin administration schedule and dosing differ between North America and Europe [4,5,6,7,8]. The North American regimen described by Packer et al. in 1993 has a 12-week induction, carboplatin 175 mg/m^2^ weekly for 4 weeks, followed by a 2-week break and then continued for 4 more weeks. Vincristine at a dose of 1.5 mg/m^2^ (max 2 mg) was concurrently administered for 10 weeks, followed by 12 maintenance cycles consisting of weekly carboplatin and vincristine, weekly for 3 weeks out of 6-week cycles [5]. The European style of CV was initially described by Gnekow et al. in 2004, is a 10 week induction, carboplatin 550 mg/m^2^ every 3 weeks for four doses with concurrent weekly vincristine 1.5 mg/m^2^ (max 2 mg), followed by 10–6 week continuation cycles with carboplatin in week 1 and vincristine in weeks 1–3 [9]. Despite these differences, results have demonstrated similar 5-year PFS of 39–47% [5,9].

Carboplatin hypersensitivity reactions remain a significant challenge to CV regimen application and have been reported in up to 7–53% [7,10]. Several groups report the possibility of a desensitization protocol consisting of antihistamine and corticosteroid premedication, followed by an incremental increase in carboplatin [11]. Carboplatin has also been used as monotherapy in PLGG; 3-year PFS was reported at 64% and 5-year PFS at 51% [12,13], although, as in the CV regimen, hypersensitivity reactions limited this regimen.

Other regimens have been studied as alternatives to the standard combination of carboplatin-based regimens. The COG A9952 clinical trial randomized the CV compared to thioguanine, procarbazine, CCNU, and vincristine (TPCV). The 5-year PFS was at 39% for patients who received CV and at 52% for patients who received TPCV. However, this difference was not statistically significant (*p* = 0.10) [6]. Toxicity was moderately higher in the TPCV arm, and there was also concern of possible secondary neoplasms, although none were reported in the participants with a median follow-up period of 5.7 years. Hence, TPCV is considered the second-line therapy after CV regimens. Weekly vinblastine has been studied for first-line therapy, along with second-line therapy. In a prospective trial, the 5-year PFS was 53% when used as the first-line therapy [14] and 43% when used as the second-line therapy [15]. Overall, the toxicity of vinblastine was minimal, with the most frequent toxicity being hematologic. Due to low toxicity and good tolerance, vinblastine has been the first-line chemotherapy regimen preferred in many institutions. Several other regimens have been evaluated for PLGG, including vinorelbine, cisplatin, etoposide, and temozolomide [16,17,18,19]. Despite different combinations and regimens, the response rates are comparable. The choice of first-line or second-line therapy is guided by favorable toxicity, tolerance, and late sequelae [3]. Since 2008, multiple molecular driver alterations, mainly within the MAPK pathway, have been identified in PLGG. It remains to be fully understood which molecular subgroups derive greater benefits from conventional chemotherapy vs. targeted therapy from MAPK kinase pathway inhibitors. There are currently multiple clinical trials (NCT06381570, NCT05566795, NCT04166409, NCT06666348, NCT04576117, NCT04485559, NCT03871257, NCT05180825) investigating these targeted agents either against a conventional chemotherapy regimen or in combination with chemotherapy, and results of these studies may alter the primary role for chemotherapy in PLGG in the future.

## 3. Molecular Characterization and Tailoring Chemotherapy Intensity in Pediatric Medulloblastoma

In addition to the exploration of small-molecule targeted agents in the treatment of pediatric CNS tumors, a deepened understanding of molecular genetics has led investigators to use this knowledge to decrease the use of chemotherapy to reduce lasting toxicities, if possible, while maintaining adequate curative outcomes. On the other hand, cure rates for some disease molecular subtypes are inadequate, and thus, intensification of treatment is also required. Medulloblastoma (MB), being the most common malignant brain tumor of childhood [20], has become the prototype CNS tumor, to gain biological understanding with the goal of directive therapy. Molecular characterization of MB using various next-generation sequencing techniques, transcriptome studies, and DNA methylation studies has classified MB now into four subgroups, WNT, SHH, Group 3, and Group 4 [21,22,23,24,25,26]. Risk stratification of patients was traditionally based on histological classification and clinical factors such as age, metastatic status, and extent of resection. Incorporating molecular classification into MB risk stratification has led to a decrease in therapy in WNT MB and the use of carboplatin as a radiosensitizer in Group 3 MB.

### 3.1. De-Escalating Therapy in WNT-Medulloblastoma

Excellent survival outcomes of WNT-MB have been reported in multiple retrospective series and prospective clinical trials (HIT 2000, SJMB03, COG ACNS 0331, COG ACNS 0332) with 5-year PFS of 80–100% and 5-year OS of 80–100%, even in the context of incomplete resection and or metastatic disease [27,28,29,30] (Table 1). However, treatment-related toxicities, including four late deaths, were reported in the SJMB03, with one patient who developed pulmonary fibrosis and three patients who died of secondary malignancies [27]. Due to the excellent outcomes overall for patients with WNT-MB, the most recent therapeutic clinical trials are investigating a reduction in radiotherapy and chemotherapy (cyclophosphamide, cisplatin, vincristine) doses.

Based on the preliminary results of the St. Jude clinical trial SJMB12, patients with WNT-MB with M0, R0, and monosomy 6 could possibly be treated with a significant reduction in CSI to 1500 cGy and 5100 cGy primary site boost followed by only four cycles of chemotherapy resulting in cumulative doses of 8 mg/m^2^ vincristine, 300 mg/m^2^ cisplatin and 12 mg/m^2^ cyclophosphamide [32]. In parallel, the recently closed COG ACNS 1422 (NCT02724579) evaluated the same population, irrespective of monosomy 6 status, with a reduced dose of CSI to 1800 cGy and a limited target volume boost of 3600 cGy to the tumor bed while eliminating vincristine during RT and reducing vincristine dosing during maintenance. The European clinical SIOP PNET-5 similarly reduced CSI in this population to 1800 cGy, as well as the number of adjuvant chemotherapy cycles. Until the results of these trials are published, discussing dose reduction with families will remain a challenging conversation. However, it is a discussion that may become routine, as reduced therapy—with lower doses of CSI and less adjuvant chemotherapy—appears poised to become the new standard of care for low-risk WNT medulloblastoma.

### 3.2. Radiosensitization in Medulloblastoma

Radiation therapy in medulloblastoma continues to be a mainstay of therapy, particularly in high-risk disease. The use of radiosensitizing agents has been evaluated to increase efficacy and improve disease-free survival. Platinum agents, specifically carboplatin, are known to cross the blood–brain barrier, be present in unbound form, enhancing the production and persistence of single- and double-strand breaks in DNA [37]. In the CCG 99701 trial, 161 patients with high-risk medulloblastoma or supratentorial primitive neuroectodermal tumors were non-randomized to the addition of carboplatin, along with vincristine concurrently with RT. The use of carboplatin as a radiosensitizer was found to be safe and feasible [38]. Subsequently, in the COG study, ACNS0332 evaluated the impact of carboplatin as a radiosensitizer in addition to vincristine during RT in patients with metastatic disease, incompletely resected MB, or with large-cell anaplastic histology. No significant difference in survival was detected for the entire cohort between the two arms. However, in post-hoc analysis, the group 3 MB derived survival benefit from carboplatin compared to other molecular subgroups, with a 5-year PFS of 73.2% with carboplatin vs. 53.7% without (*p* = 0.047) [28]. Based on these results, it is now recommended to consider the addition of concurrent carboplatin during RT in high-risk group 3 MB. While this study’s findings indicated an incremental gain in survival for the high-risk group 3 MB, this analysis was based on a small number of patients, and it remains unclear whether additional molecular markers, such as the *MYC* amplification or isochromosome 17, may have contributed to the observed difference. However, based on the findings of ACNS 0332, COG and other consortiums are considering including carboplatin in the successors’ studies [39]. This introduces ongoing controversy due to the interim addition of incorporating molecular classification of MB within the analysis while the trial is ongoing, but on balance, it also recognizes the significantly inferior outcomes of the metastatic group 3 MB patients. Ideally, incorporation of carboplatin prospectively in future confirmatory clinical trials will be helpful in establishing true practice-changing recommendations.

Chemotherapy continues to be an essential component of multimodal therapy in MB. Despite success in de-escalation of therapy in WNT-MB, novel therapies or intensification of therapy are required in certain subgroups, such as SHH with *TP53*-mutant or *MYCN* amplification, Group 3 with *MYC* amplification, or metastatic Group 3 and 4. The role of carboplatin continues to be questioned, but ultimately, there is a need to balance exposing children to toxic side effects while trying to improve survival in a disease that is not salvageable yet at relapse.

## 4. Intensification of Chemotherapy to Avoid or Delay Radiotherapy in Young Children with Embryonal CNS Tumors

Very young children, ages 0–5 years, have long been identified to pose a unique therapeutic challenge and continue to be. Craniospinal irradiation (CSI) is typically not recommended due to the devastating neurocognitive sequelae associated with its use. This limitation resulted in multiple working groups to develop chemotherapy-only or radiation-delaying chemotherapy protocols in the hope that a child can be older and achieve more brain development prior to irradiation (Table 2). The Pediatric Oncology Group (POG), CCG, Baby Brain French Society of Pediatric Oncology (BBSFOP), and German Therapieprotokoll für Säuglinge und Kleinkinder mit Hirntumoren (HIT-SKK) groups all implemented approaches that involved delaying radiotherapy (e.g., POG 8633/34, also known as Baby POG-1, CCG 921, BBSFOP, HIT-SKK’87, and HIT-SKK’92 trials).

In the Baby POG-1 trial, children under 3 years old were treated with chemotherapy, including vincristine, cyclophosphamide, etoposide, and cisplatin, with CSI administered at 1 or 2 years after diagnosis, depending on the child’s age [40]. The CCG 921 trial used an “eight-drugs-in-a-day” regimen [56]; BBSFOP used a 16-month regimen of carboplatin/procarbazine, cisplatin/etoposide, and vincristine/cyclophosphamide [45], and HIT-SKK incorporated high-dose methotrexate (HD-MTX) with alkylators and platinum therapies [46].

Given the limited success reported with conventional chemotherapy alone in this young age group [40,44,45,47,57,58], high-dose chemotherapy (HDC) was introduced about three decades ago as another alternative to CSI in the treatment of all types of malignant CNS tumors of early childhood by intensifying chemotherapy with the goal to delay, decrease, or omit radiotherapy [48,51]. Based on the limited efficacy in ependymal and glioneuronal tumors, more recent trials and published treatment have narrowed down its use to high-risk embryonal CNS tumors, such as MB, atypical teratoid rhabdoid tumor (ATRT), and other rare embryonal subtypes with recognized chemosensitivity, while accepting trade-offs and acute toxicities of HDC.

High-dose chemotherapy (HDC) approaches have been more commonly implemented as frontline treatment for CNS embryonal tumors of early childhood in North America compared to Europe [59] (Table 2). Two main North American study groups have developed HDC-based clinical trials, using two different conditioning regimens. The Head Start group [60,61] has established their experience over four consecutive trials using a single consolidation cycle of high-dose thiotepa (300 mg/m^2^/d × 3 days), etoposide (250 mg/m^2^/d × 3 days), and carboplatin (AUC of 7/d × 3 days), following an induction phase of three to five cycles of conventional chemotherapy with high-dose methotrexate (HD-MTX). The COG has pursued a consolidation phase with three sequential cycles of high-dose thiotepa (10 mg/kg/d × 2 days) and carboplatin (17 mg/kg/d × 2 days), following a usual number of three cycles of induction with or without HD MTX [48,54]. In Europe, the French Society of Pediatric Oncology (SFCE) conducted a multicenter Phase I/II clinical trial (HR MB-5) for children aged five and under with high-risk MB using two cycles of high-dose thiotepa (600 mg/m^2^) and one cycle of cyclophosphamide and busulfan for complete responders [55].

### 4.1. HDC for Medulloblastomas

Multiple studies have demonstrated the benefit of HDC for infants and young children (iMB) with desmoplastic histology (likely SHH group), showing favorable outcomes without radiotherapy, with 5-year PFS of up to 89% [36,58,60,62]. For young children <3 years with MB and other embryonal CNS tumors, the COG’s latest trial ACNS 0334 (NCT0033602) for high-risk MB and other embryonal CNS tumors used the previously described backbone of three cycles of induction (vincristine, cyclophosphamide, etoposide, cisplatin), followed by HDC with three cycles of high-dose carboplatin and thiotepa and stem cell rescue [48], but added HD-MTX in a randomized manner during induction to assess response rate and PFS. This study enrolled 39 patients, and preliminary results have been reported in abstract form, showing that, like for older patients with HR group 3 MB, intensifying therapy may be beneficial [63]. The patients randomized in the HD-MTX arm during induction had a significantly better response rate to therapy (63% versus 30% *p* = 0.038) and showed a trend toward better PFS than those who did not (2-year PFS of 68.2% +/−9.6% vs. 45.8% +/− 13.8%; *p* = 0.08). The addition of HD-MTX significantly benefited patients with group 3 MB, leading to a 5-year OS of 80% compared to 40% for the group 3 patients who did not receive HD-MTX during induction. The trial enrolled 11 patients with metastatic SHH MB, evenly distributed in the two randomized arms for HD-MTX. The survival for the SHH subgroup was excellent, achieving 100% in both arms.

While more extensive analysis is needed to evaluate the impact of additional prognostic markers such as Myc and metastatic status, this trial provides encouraging results and perspectives for some patients with group 3 MB, allowing for improved cure with limited use of adjuvant radiation following HDC. As for the patients with SHH MB, the excellent outcome in both arms questions the need for such intensive therapy for this group of patients and may suggest careful de-escalation of therapy.

In parallel, the Head Start group in its latest clinical trial, Head Start 4 (NCT02875314), compares in a randomized fashion the impact of one cycle of consolidation (carboplatin, thiotepa, etoposide) to three consolidation cycles as used in the COG protocol (carboplatin, thiotepa) for high-risk MB and other embryonal tumors. However, patients with SHH MB were considered low risk irrespective of their metastatic status and were non-randomly assigned to one single cycle of consolidation, following three to five cycles of induction with HD-MTX. The results for the 39 patients with the SHH MB arm were recently reported. The 28 patients with localized SHH achieved a 3-year PFS of 96.4% (CI 73.2–100%) compared to 36.4% (95% CI 16.6–79.5%) for the 11 patients with disseminated SHH MB. All but four (89%) patients had received three cycles of induction and one cycle of consolidation [64]. This data upon maturity and publication, in addition to the excellent outcome described in ACNS 0334 for the SHH MB, may indicate that localized SHH iMB may safely be treated with only one cycle of consolidation (carboplatin, etoposide, thiotepa) following three cycles of induction with HD-MTX, while patients with metastatic SHH are expected to achieve an excellent outcome if undergoing three cycles of consolidation (carboplatin, thiotepa), following induction with or without HD-MTX. Although such an HDC strategy is not without toxicity and long-term complications, including hearing impairment and infertility, these recent trials have further solidified the evidence that overall, SHH iMB do not require adjuvant radiation to achieve excellent cure rates, provided chemotherapy intensity is sufficient [65,66]. In addition to the success in SHH iMB in North America, the HIT SKK 2000 trial in Germany also reported excellent survival for non-metastatic SHH iMB using conventional carboplatin instead of cisplatin, and serial injection of both intraventricular and intravenous MTXIT/IV MTX with a 5-year PFS of 93% [46]. Therefore, the next interesting question in SHH iMB would be to understand whether Head Start 4, COG ACNS 0334, or HIT SKK 2000 would have comparable survival, and whether a specific regimen may possibly reduce neurocognitive toxicity. The CONNECT consortium is currently planning this type of clinical trial to potentially answer this question.

The Head Start 4 trial was recently closed to accrual for high-risk patients with non-SHH MB or other embryonal tumors. The results of the randomization of one vs. three cycles of consolidation are now awaited, to report the potential benefit of three cycles of consolidation over one and provide survival rates for the groups 3 and 4 in iMB. More refined molecular characterization within group 3, especially with Myc amplification status, may help identify further patients who fare poorly with HDC and for whom new strategies are needed. Finally, it is unclear whether young patients with group 4 iMB should continue to be grouped together with those with group 3 MB, in light of their response to HDC and different pattern of relapse [67]. In Europe, the HR MB-5 trial showed some promising results in nine (32%) of young MB children who did not require radiotherapy (median follow-up 5 years), but unfortunately, this study was prematurely stopped due to an excess of events [55].

### 4.2. HDC for Atypical Teratoid Rhabdoid Tumors

Identified in the late 1990s as a separate entity from MB and more broadly from the formally named PNET, atypical teratoid rhabdoid tumor (ATRT) is a subset of aggressive CNS embryonal tumors of early childhood characterized by the loss of expression of INI by immunohistochemistry (IHC), which were, until recently, associated with a very dismal prognosis [68,69]. Early European and North American trials using the standard “infant embryonal brain tumor approach”, with conventional chemotherapy only, have yielded dismal PFS under 20% [43,44]. The COG trial ACNS 0333 was the largest prospective clinical trial specifically dedicated to ATRT based on HDC. The protocol included two cycles of induction with HD-MTX and consolidation with three cycles of high-dose carboplatin and thiotepa, but also involved age-based RT for all patients. This multimodal approach led to significant improvement in survival compared to historical controls, bringing up the 4-year PFS and OS to 37% and 43%, respectively, while reporting a toxicity-related mortality of 6%. The trial also retrospectively allowed for the molecular characterization of the cohort according to the consensus molecular subgrouping of ATRT [70]. The SHH-NOTCH, TYR, and MYC subgroups accounted for 30.3%, 42.8%, and 26.7%, respectively. Survival by molecular subgroup (4-year OS) was 56%, 41%, and 27% for the SHH-NOTCH, TYR, and MYC subgroups, respectively. While patient numbers were too small in each group to detect a significant difference, the survival pattern was in accordance with the previous retrospective reports, whereby SHH-NOTCH might represent a more responsive group to therapy [54]. While adjuvant RT has been associated with a survival benefit [71], a limited series of patients with ATRT treated with a multimodality approach using HDC have described long-term survivors spared from adjuvant RT. In the Canadian ATRT registry experience, 11 of the 18 patients treated with HDC did not receive adjuvant RT. Six of 12 survivors were treated with HDC without RT [72,73]. Although these limited experiences, including small sample sizes, indicate that survival without RT is possible, the exact characterization of such patients still needs to be delineated. The combination of molecular subgrouping with clinical and therapeutic factors may help in defining these patients in future trials. Conversely, the HDC strategy may not be the ultimate approach for some patients with ATRT, as suggested by the poor survival on the ACNS0333 trial, of 26.7% for the MYC subgroup, which remains close to historical control survival.

A better understanding of the molecular landscape of these three subgroups and investigations of their distinct therapeutic vulnerabilities may lead to more specific targeted therapies [70,74]. All future efforts to raise survival for patients with ATRT should be a high priority, due to the very young age of the patients at diagnosis and their extreme susceptibility to cranial RT.

### 4.3. HDC for Embryonal Tumor with Multilayered Rosettes

The new entity of embryonal tumors with multilayered rosettes (ETMR) entered the WHO classification of CNS tumors in 2016, to regroup under the same umbrella of CNS embryonal tumors alongside previous entities such as embryonal tumor with abundant neuropil and true rosettes (ETANTR), medulloepithelioma, ependymoblastoma, and CNS-PNET, which shared recurrent amplification or gene fusion of C19MC, an oncogenic microRNA on chromosome 19 [75,76,77]. Some limited data are becoming available on the impact of HDC for this rare and aggressive entity. In the series of 159 patients from the Rare Brain Tumor Registry, Khan et al. described adverse outcomes associated with incomplete resection, metastatic presentation, and brainstem location. HDC was identified both in univariate and multivariate analyses, as a favorable prognostic criterion for survival [78]. Similarly, in the population-based prospective P-HIT trial, Juhnke et al. reported 18 patients with ETMR treated with induction chemotherapy, tandem consolidation with high-dose carboplatin, and etoposide, followed by high-dose cyclophosphamide and thiotepa. Adjuvant RT was not intended for patients in complete remission (CR) before HDC and was optional for patients who achieved CR after consolidation with HDC. The 5-year PFS and OS for the 17 patients with non-brainstem ETMR who received HDC were 35% and 47% compared to 0% and 8% for the 13 who received non-HDC-based therapy. As previously reported for ETMR, a significant percentage of patients rapidly progressed during induction chemotherapy. Three of the five patients (60%) who completed their consolidation with HDC were radiation-free survivors, while all four patients who received RT after HDC consolidation were alive [79].

The SFCE reported their retrospective experience on 38 patients with ETMR, of whom 30 were treated with HDC with various combinations of tandem consolidation cycles using drugs, including melphalan, cisplatin, and thiotepa. Thirteen of the patients treated with HDC (43.3%) also underwent RT, either in an adjuvant setting or at the time of progression. At last follow-up, 11 patients were reported alive, including 8 of those treated with HDC (26.6%). In their analysis, the use of HDC was associated both in univariate and multivariate analyses with better OS [80].

The prospective COG trial, the ACNS0334 trial, enrolled 14 patients with ETMR. Patients largely presented with localized disease at diagnosis, and a third progressed early on during therapy. Four patients (28.5%) were alive without progression at 5 years of follow-up, and none of them had received RT. Disease progression or relapse was essentially local (88%). Four patients received salvage RT at the time of recurrence or progression, and all died from disease [63]. Altogether, these data suggest that consolidation with HDC may benefit a portion of ETMR but does not address the early progression, often occurring prior to consolidation. Given the propensity of these tumors for local relapse, local control with early focal RT may be an important component of future trials [81].

## 5. Adjuvant Chemotherapy to Reduce Radiation Field and Doses in Central Nervous System Germ Cell Tumors (CNS GCTs)

Central nervous system GCT represents another subgroup of CNS malignancies that have achieved improved PFS, whose goal of past, present, and future planned clinical trials focuses on therapy reduction, especially in the reduction in radiation field and doses, with the use of neoadjuvant chemotherapy in hopes of reducing long-term neurologic sequelae.

### 5.1. Germinoma

Overall survival in germinomas exceeds 90% in retrospective and prospective series [82,83,84,85]. Historically, only CSI has been the gold standard treatment for CNS germinoma [86,87,88]. Adjuvant systemic chemotherapy was introduced with the aim of reducing radiation doses and/or volume while maintaining high cure rates [89,90]. Chemotherapy-only approaches were attempted in the First, Second, and Third International CNS Germ Cell Tumor Studies but led to unacceptable high recurrence rates with PFS ranging between 40 and 50% [91,92,93,94,95,96], and thus, the current treatment goals are balancing the minimum amount of radiation required in combination with chemotherapy.

Germinomas are highly sensitive to chemotherapy, particularly to platinum compounds and cyclophosphamide [97,98]. The international groups, including SIOP (International Society of Pediatric Oncology, SIOP-CNS-GCT-96 protocol), French SFOP, and Japanese working groups, had set the foundation in replacing CSI with a reduced field radiation to whole ventricular radiation with the addition of chemotherapy with carboplatin, etoposide, and ifosfamide combinations. The major learnings from these successive trials were the rare ventricular relapses in patients who received focal radiation and chemotherapy, which helped establish the now-standard approach in Japan, Europe, and North America of combining chemotherapy with whole ventricular radiation (WVI). It is still not clear what optimal dose of the focal boost is required or how low the dose of WVI can be administered without significant rates of relapse, but upcoming clinical trials continue to explore this question. The most recently published Children’s Oncology Group Trial ACNS 1123 evaluated the efficacy of a reduced dose of 1800 cGy WVI (plus boost to tumor bed to a total of 3000 cGy) in germinoma for patients with a complete response to chemotherapy, resulting in an excellent 3-year estimated PFS rate of 94.5 +/−2.7% [82]. Although the reduced radiation dose of 1800 cGy was short of the statistical noninferiority design using a 3-year PFS rate of 95%, the estimated PFS was still favorable, and neurocognitive evaluation showed that this cohort had a lower mean attention score at 9 months compared to those who received 2400 cGy and improvement in processing speed at 30-month assessment [82].

Among the variety of chemotherapy regimens, no protocol has suggested better activity or improved outcome. Given that the goal is to optimize short- and long-term safety of the chemotherapy used, both the Japanese and North American working groups have consistently used the combination of carboplatin and etoposide, which can be delivered as an outpatient over 3 days and also does not require hyperhydration that could cause electrolyte imbalance in this at-risk population already often managing diabetes insipidus [99].

### 5.2. Non-Germinomatous Germ Cell Tumors (NGGCTs)

NGGCTs are a group of diverse tumors that require differing treatment approaches compared to germinoma. Despite this, recognizing the importance of preservation of long-term neurological function and reduction in late side effects, a similar approach to that used with germinomas to reduce the reduction in field and dose of radiation has been employed with the use of multimodal treatment with surgery and chemotherapy. Unlike germinoma, NGGCTs are relatively non-responsive to RT alone, with reported 5-year survival ranging from 20 to 40% [100]. In addition, chemotherapy alone has only a modest effectiveness in NGGCTs. Typical agents used include carboplatin, cisplatin, cyclophosphamide, ifosfamide, and/or etoposide [101]. The optimal volume of radiation therapy in the context of neoadjuvant chemotherapy is still a matter of debate. SIOP protocols recommend focal irradiation for nonmetastatic NGGCT, while North American groups traditionally used CSI. Five-year PFS were at 72% and 84%, respectively, for the SIOP-CNS-GCT-96 and COG ACNS0122 studies, but CSI exposed patients to substantial risk of long-term side effects [102,103,104]. Therefore, the COG clinical trial ACNS 1123 attempted to evaluate the reduction in RT to 3060 cGy whole ventricular field and 5400 cGy tumor bed boost in patients with localized NGGCT who achieved a complete or partial response with or without second-look surgery. Eight of 66 (12.1%) patients eligible for reduced radiation subsequently progressed, with six of them presenting with distant spinal relapse and two having combined local and spinal relapse. Although PFS was similar to the previous COG study, ACNS0122, with a 3-year PFS of 87.8%, the treatment failure pattern to the spine was concerning, although some children with relapse did not have normalization of serum tumor markers but remained under study and may have incorrectly received de-escalated therapy [105]. Nonetheless, the currently open COG clinical trial CNS 2021 (NCT04684368) added spinal canal irradiation to the WVI approach.

## 6. Challenges and Perspectives

Although much progress has been made in optimizing the benefits of conventional chemotherapy in the treatment of pediatric malignant brain tumors, there remain limitations in its use, specifically with drug efficacy in certain subtypes of CNS tumors, relapsed disease, and limitations of effective drug delivery to the brain.

### 6.1. Unknown Role for Conventional Chemotherapy

#### 6.1.1. Ependymoma

Curative therapy with the use of chemotherapy remains a challenge for many other pediatric CNS tumor types. For ependymoma, the third most common brain tumor in children, history is fraught with negative trials for the use of many chemotherapeutic agents [106]. The role of chemotherapy in ependymoma has been investigated in clinical trials with three different purposes: (1) to improve survival following surgical resection and radiotherapy, (2) to facilitate second-look surgery after incomplete resection, and (3) to delay or avoid radiotherapy in young children. The recently closed COG trial ACNS0831 (NCT01096368) investigated, in a randomized manner, the potential benefit of maintenance chemotherapy for completely resected ependymoma following focal RT. Preliminary results were presented in abstract form, describing the absence of a significant difference in 3-year PFS for patients who received chemotherapy vs. those who received RT only (78% vs. 72%). While the trial suffered significant noncompliance with randomization to post-RT chemotherapy in about 30% of the patients, there was no observed benefit to maintenance chemotherapy [107,108]. An older COG trial, ACNS 0121, studied a group of children who received adjuvant chemotherapy to facilitate second-look surgery to increase the rate of GTR in children with initial complete resection. Second-look surgery was undertaken in only 39%, and among them, 56% achieved GTR [109]. To delay or avoid RT in young children, the CCG 9233/34 trial compared an intensive dose to a standard dose of adjuvant chemotherapy in young children with all malignant CNS tumors. The United Kingdom Children’s Cancer Study Group (UKCCSG) and SIOP group used methotrexate-based chemotherapy for children 12 months to 5 years old, and Head Start studies added HDC. These intensive regimens reported 2-year PFS rates of around 40% or less but did not influence overall survival, as still only 40% of survivors were spared from RT [44,110,111].

Our growing understanding of the molecular landscape of pediatric ependymomas, posterior-fossa A ependymoma, posterior fossa B ependymoma, supratentorial ependymoma, *ZFTA* fusion-positive (formerly called *RELA* fusion-positive), supratentorial ependymoma, and *YAP1* fusion-positive [22] has helped refine prognostic factors but has not yet impacted treatment strategies. However, recent in vitro studies have indicated a significant reduction in cell proliferation in a subtype of high-risk ependymoma of the posterior fossa defined as Posterior Fossa A Ependymoma (PFA) with chromosome 1q gain when exposed to RT and the combination of 5-fluorouracil (5-FU) and All-trans retinoic acid (ATRA), whereby 5-FU acts as a radiosensitizer. Previous single-agent phase I studies for 5-FU and ATRA are available for the pediatric population, and this should facilitate the phase II investigation of this combination in relapsed high-risk PFA soon.

#### 6.1.2. Pediatric High-Grade Glioma

Despite years of extensive research, no specific chemotherapy regimen has proven to be particularly effective in pediatric high-grade glioma (PHGG), and there is no established standard chemotherapy treatment for children [112]. Chemotherapy was first demonstrated to provide a potential survival benefit in 1976, when studies led by CCG (CCG-943 and CCG-945) found that combining radiation therapy with lomustine, vincristine, and prednisone (PCV) or eight-in-one drugs improved survival compared to radiation therapy alone [113]. However, it was also noted that chemotherapy remained ineffective for patients with unresected tumors [114]. In 2005, Stupp et al. reported that using adjuvant temozolomide after radiotherapy significantly prolonged survival among adult patients with newly diagnosed glioblastoma, with a median increase in survival of 2.5 months or a relative reduction in the risk of death of 37 percent. At two years, they found a clinically meaningful increase in the survival rate, from 10 percent with radiotherapy alone to 27 percent with radiotherapy plus temozolomide [115].

Following this, the Children’s Oncology Group (COG) conducted a phase 2 trial (ACNS0126) to evaluate a similar treatment regimen in children, using a higher dose of temozolomide (90 mg/m^2^/day versus 75 mg/m^2^/day) during radiation. The results of the pediatric trial showed 1-year EFS and OS rates similar to those seen in adults, but the difference was not statistically significant [116]. The successor COG trial, ACNS0423, intensified treatment by using an additional second active alkylating agent, lomustine. This results in a significantly greater 1-year EFS compared to ACNS 0126 of 49% vs. 37%, a 3-year EFS 22% vs. 11%, and a significantly different 3-year OS of 28% vs. 19% [117].

Recent advancements in sequencing technologies have made it possible to analyze large numbers of tumor samples at the molecular level. By combining DNA and RNA sequencing with proteomics and methylation analysis, researchers have classified PHGG into distinct subgroups: H3-mutant (K27 altered or G34 mutant), IDH-mutant, H3/IDH-wildtype (WT), and infant-type hemispheric glioma [22,118]. This classification has opened opportunities to explore chemotherapy agents that can specifically target these unique molecular characteristics (Table 3).

Advancements in understanding the molecular biology of pediatric CNS tumors have allowed for exploration of targeted approaches, including agents targeted at specific cancer mutations, histone modifications, and even a variety of uses of immune-targeted therapies. These innovative treatment approaches beyond standard chemotherapeutic agents are likely needed to achieve better outcomes and are currently being explored in early-phase clinical trials.

#### 6.1.3. Role of Chemotherapy in Other Rare CNS Tumors

Chemotherapy may continue to have a small but important role in some rare CNS pediatric tumors. Choroid plexus carcinomas (CPC) seem to benefit from alkylator chemotherapy regimens using backbones containing ifosfamide, carboplatin, and etoposide (ICE), with 5-year PFS ranging from 50 to 60% with various regimens [119,120]. In addition, survival has been reported to be superior in patients with wild-type TP53 (WT) tumors rather than TP53-altered (*p* = 0.012) [121]. Like other young patients with malignant tumors, HDC has been tried in small cohorts to avoid/delay RT with RT-free survival rates of about 50% both in retrospective reports and prospectively in the Head Start experience [120,122]. A better understanding of how germline TP53 impacts chemotherapy response will help tailor future therapy. A current clinical trial in development through the Pediatric Neuro Oncology Consortium (PNOC) will allocate treatment based on TP53 status where patients with WT TP53 CPC will receive conventional ICE chemotherapy with or without focal RT and maintenance therapy, while patients with altered TP53 tumor or with germline mutation (high risk) will undergo consolidation with HDC followed by maintenance chemotherapy without adjuvant RT [Lafay-Cousin personal communication].

Craniopharyngioma (CP) is another rare tumor that has seen some possible benefit with the use of medical therapy to delay RT in the very young child. While these tumors are generally not highly aggressive beyond their local area, their treatment is difficult due to their proximity to vital structures around the sella, such as the visual pathways, pituitary gland, and hypothalamus [123,124,125]. Young children typically have the adamantinomatous (cystic) subtype [126], and standard treatment usually involves careful surgical removal and possibly localized RT. This is particularly challenging because the CP and this treatment often lead to long-term health complications, including significant hypothalamic and pituitary dysfunction [127].

Systemic chemotherapy has limited efficacy in treating CP. Based on a promising case series [128], subcutaneously administered pegylated interferon was prospectively studied in the Pediatric Brain Tumor Trial Consortium PBTC-039 trial (NCT01964300) in progressive or recurrent pediatric patients, with, unfortunately, no objective responses observed [129]. Intracystic therapy has also been used in cystic CP. During each treatment, a reservoir implanted surgically within the tumor cyst is accessed with a needle; cyst fluid is withdrawn, and the same volume of fluid is replaced with the treatment agent. One of the first agents tested in this way was intracystic bleomycin, which showed positive results in shrinking or stabilizing cysts. However, patients experienced significant side effects, particularly neurotoxicity from bleomycin leakage, which caused complications such as meningitis, cranial neuropathies, major hypothalamic damage, seizures, blindness, fever, headaches, nausea, vomiting, and even death [130,131]. Intracystic interferon-alpha therapy (Roferon, Intron A) offered a more favorable side effect profile. The rationale for using interferon is based on the idea that the cyst walls of CP tumors share similarities with the cells of squamous cell skin carcinomas, which are known to respond to interferon’s antiproliferative and immunomodulatory effects. The most common side effects (typically mild to moderate) include flu-like symptoms, headaches, fatigue, transient hyponatremia, appetite loss, and weight loss. Intracystic treatment does not affect the solid component of the tumor and is solely beneficial to the cyst being treated. Treatment is often considered in the setting where a child has a symptomatic cystic-predominant CP, with the hopes of delaying primary surgical treatment or radiation that can lead to later morbidities. While no prospective studies have been conducted on this treatment, a cohort study in children showed clinical and radiologic improvement in 47 out of 60 patients (78.3%) treated with intracystic therapy [132]. Unfortunately, the formulation used internationally is no longer available globally, and pegylated interferon is now being considered for future evaluation as an alternative for intracystic treatment.

### 6.2. Chemotherapy in Relapsed CNS Disease

Attempts at salvage therapy for malignant CNS tumors with chemotherapy remain limited.

Some patients with localized relapse may undergo repeat surgery and focal radiation. Institutional experiences of re-irradiation with or without chemotherapy have shown prolonged survival mainly for relapse in average-risk MB patients [133,134]. Given the limited life expectancy after relapse following CSI, regimens of lower doses of metronomic chemotherapy have been used to prolong life, but a sustained survival benefit was only reported recently. The Medulloblastoma European Multitarget Metronomic Anti-Angiogenic Trial (MEMMAT) was a phase 2 multicenter prospective trial that enrolled 40 patients with relapsed MB. The regimen includes daily oral fenofibrate, thalidomide, and celecoxib and alternates low dose every 21 days of etoposide, cyclophosphamide, along with bevacizumab and serial injection of intraventricular etoposide and cytarabine. Treatment duration was 12 months with possible extension without etoposide or cyclophosphamide and with extended intervals of intraventricular therapy, depending on response and tolerability. With this regimen, 23 (57.5%) achieved disease control after 6 months, and response was detected in 45%. The 3- and 5-year PFS were both at 24.6%, indicating prolonged tumor control, especially for the 18 patients demonstrating tumor response who had a 5-year PFS of 49.7% [135]. The COG ACNS 0821 was a phase 2 study for relapse MB or PNET, evaluating in a randomized manner the addition of bevacizumab to the irinotecan-temozolomide drug combination. Irinotecan was given for 5 days IV concomitantly with oral temozolomide, while bevacizumab was administered on day 1 and 15 of each 28-day cycle. This study accrued 105 patients. Patients who received bevacizumab had a better survival than those who did not, with a median PFS of 9 months for the bevacizumab arm and 6 months for the arm without bevacizumab. The median survival time was also significantly better for those who received bevacizumab at 19 months compared to 13 months. Twenty-three percent of the patients completed 12 cycles, and the toxicity profile was comparable in both arms [136]. While none of these regimens can be presented as a cure to families, they provide an extension of survival with a reported acceptable toxicity profile.

### 6.3. The Ongoing Stumbling Block of Overcoming the Blood–Brain Barrier

Obstacle: The blood–brain barrier (BBB) is a semi-permeable structure that surrounds the blood vessels of the central nervous system (CNS) that has always posed a challenge for chemotherapeutics. Within the capillaries, endothelial cells form tight junctions, and along with a variety of receptors, transporters, efflux pumps, and other cellular components, movements of substances between the blood and brain are heavily regulated. The BBB serves an important purpose in preventing harmful substances from reaching the brain, but it also limits the passage of over 98% of small-molecule drugs and completely restricts large-molecule therapeutics from accessing the brain [137]. For most chemotherapy drugs, the amount of the drug that reaches the cerebrospinal fluid (CSF) after a systemic dose is typically less than 10% of the total systemic exposure. As a result, intravenous chemotherapy is generally not as effective for treating CNS tumors [138].

#### 6.3.1. Intrathecal and Intraventricular Chemotherapy

Intrathecal (IT) chemotherapy delivered by lumbar puncture is the most common CNS-directed therapy used in children and accepted as an essential treatment for pediatric acute leukemia for decades [139]. Drugs available for IT delivery in pediatric oncology include methotrexate (MTX), cytosine arabinoside (Ara-C), corticosteroids, thiotepa, busulfan, etoposide, topotecan, 6-mercaptopurine, mafosfamide, and rituximab. The pharmacokinetics of IT MTX are different from IV infusions. MTX clearance is provided by CSF reabsorption and is dependent on CSF flow. MTX can diffuse from CSF to the plasma compartment, and, thus, repeated administration may mirror systemic IV infusions and cause systemic toxicity [140]. Although the CSF concentration of MTX is 100 times higher than plasma after intralumbar infusion, the CSF ventricular concentration is only about 10% of the lumbar concentration [138]. This led to studies that hypothesized intraventricular administration of MTX would provide a higher CSF concentration of the drug. Consequences of this type of chemotherapeutic delivery include the potential for chemical arachnoiditis and acute or chronic neurotoxicity. Despite common use in treating pediatric leukemia, it is not a common approach in the treatment of pediatric CNS tumors in North America. European protocols have adopted this approach more with the incorporation of serial intraventricular injections of MTX in the treatment of CNS embryonal tumors via Ommaya reservoir [46,141]. Other agents that have been used in recurrent pediatric medulloblastoma include IT etoposide and liposomal Ara-C in combination with a metronomic combination therapy that has shown the most impressive 3-year PFS of 42.0 +/−9.5% [142].

#### 6.3.2. Other Strategies

A number of other strategies to overcome the BBB have been explored, including trials using focused ultrasound-mediated drug delivery [143,144,145,146,147,148] and convection-enhanced delivery [149,150,151,152]. In other disease settings and adult HGG, additional strategies include different directed deliveries like intranasal, intra-arterial, or intracavitary chemotherapy. Use of nanoparticles with different compositions that could creatively bypass the BBB using endocytosis, receptor-mediated transcytosis, enhanced permeability and retention, or even extravasate to slowly release into tissue has also been explored [138]. Future continued clinical studies incorporating new technological approaches with the use of chemotherapeutic agents are needed to move toward curative, safe, and less toxic therapies.

### 6.4. Limitations

This review is intentionally limited in scope, focusing on select key topics related to chemotherapy for pediatric brain tumors that hold current clinical relevance, rather than providing an exhaustive systematic review of the field. Notably, this article does not address other emerging therapeutic modalities such as small-molecule inhibitors, immunotherapies, or advanced targeted drug delivery technologies, as its primary objective is to concentrate specifically on chemotherapy approaches. Furthermore, direct comparisons of response rates, event-free survival, and overall survival across the various clinical trials and studies referenced are challenging due to heterogeneity in study designs, differing primary endpoints, inconsistent reporting methods, and the typically small patient cohorts involved. Finally, given that this work is a narrative review reflecting expert opinion rather than a systematic review, it does not encompass all available international clinical trials, instead highlighting several major studies to provide representative insights into current chemotherapy treatment paradigms. Future studies would benefit from standardized reporting criteria and harmonized endpoints to facilitate more reliable comparisons across clinical trials. International collaborations are essential to increase patient numbers and improve the statistical power of outcome analyses.

## 7. Conclusions

Advances in molecular understanding of pediatric CNS tumors and the introduction of non-chemotherapy treatments like small molecule agents and different immune strategies have garnered excitement in the scientific community due to the possibilities for new and innovative therapies for children, with some hopes to improve survival in those with historically poor outcomes or decrease long-term treatment-related toxicities. However, in the present day, there continues to be a predominant role for the use of chemotherapy as the first-line treatment of many pediatric CNS tumors. Despite the equipoise in what chemotherapy agents are most effective in PLGG, it remains the first-line treatment after surgery for most, as we await the results of the head-to-head phase 3 clinical trials between chemotherapy and MEK inhibition. In CNS embryonal tumors, the use of chemotherapy has been generally moving toward intensification with increasing chemotherapy intensity to try to achieve better outcomes with the addition of radiosensitizing agents and the use of high-dose chemotherapy. Where it has become clearer that chemotherapy will not benefit are in the CNS tumors that historically had poor response rates, like ependymoma and HGG, although this was painfully established after years of many successive negative clinical trials.

Future trials using chemotherapy should really focus on pediatric CNS embryonal tumors known to be chemosensitive but for whom the ideal combination has not yet been identified, specifically MYC-amplified group 3 MB, recurrent MB, and young children with radiation-sparing approaches. Combination chemotherapy and small-molecule inhibitor trials for low-grade glioma are already ongoing, and we will soon learn about the efficacy and toxicity of this approach. Discovery of new agents is needed for other tumor subtypes, where the role of chemotherapy remains unclear.

Undoubtedly, chemotherapy will still be the mainstay of pediatric CNS tumor therapy. However, growing biological insight will allow for the selection of patients who may benefit from additional agents, and hopefully, technology will increasingly allow for the modification of directed drug delivery and immune-modulating approaches to enhance conventional approaches and improve survival in the future.

## Figures and Tables

**Table 1:** Chemotherapy and radiotherapy treatment in medulloblastoma clinical trials (adapted from Choi, 2023 [31]).

	M, R	Radiation Dose and Field	Chemotherapy Reduction (Cumulative Doses)	Preliminary Outcomes	Reference
HIT-2000	M+	Hyperfractionated CSI 4000 cGy, +800 cGy tumor boost, +2000 cGy PF boost, +1000 cGy spinal metastases boost, +2800 cGy supratentorial metastases boost Total: 6800 cGy	Pre-RT: 2 cycles: MTX intraventricular 48 mg CPM 4800 mg/m^2^ MTX IV 15 mg/m^2^ CBP 1200 mg/m^2^ VCR 9 mg/m^2^ ETOP 900 mg/m^2^ Maintenance 4 cycles: CDDP 280 mg/m^2^ CCNU 300 mg/m^2^ VCR 18 mg/m^2^	5-yr EFS 62% WNT: 5-yr EFS 100%	[30]
COG ACNS 0331 Standard dosing	M0, R0	PFRT vs. IFRT boost 5400 cGy 2340 cGy CSI vs. 1800 cGy CSI for patients aged 3–7 years	Concurrent VCR 9 cycles: (AABAABAAB) VCR 45 mg/m^2^ CDDP 450 mg/m^2^ CCNU 450 mg/m^2^ CPM 6 g/m^2^	5-yr PFS 81.4% WNT 5-yr PFS 93.3%	[29]
COG ACNS 0332 Standard dosing	M+, R+	3600 cGy CSI 5580 cGy PF boost	Concurrent randomized Regimen A: VCR 9 mg/m^2^ Regimen B: VCR 9 mg/m^2^ CBP 1050 mg/m^2^ Regimens A and B, 6 cycles: VCR 18 mg/m^2^ CDDP 450 mg/m^2^ CPM 12 g/m^2^	5-yr PFS 62.9% CBP 66.4% vs. No CBP 59.2% (*p* = 0.11) WNT: 5-yr PFS 92.9% Group 3: 5-yr PFS CBP 73.2% vs. No CBP 53.7% (*p* = 0.047)	[28]
COG ACNS 1422 Reduction	M0, R0	1800 cGy CSI 5400 cGy PF boost	7 cycles (ABABABA) VCR 27 mg/m^2^ CDDP 300 mg/m^2^ CCNU 300 mg/m^2^ CPM 6 g/m^2^	Active, not recruiting	NCT02724579
SJMB03 Standard dosing	AR: M0, R0 HR: M+, R+	AR: 2340 cGy CSI 5580 cGy PF boost HR: 3600 cGy (M0/M1), 3960 cGy (M2/M3) CSI 5580–5940 cGy PF boost	4 cycles VCR 8 mg/m^2^ CDDP 300 mg/m^2^ CPM 16 g/m^2^	AR 5-yr PFS 83.2% HR 5-yr PFS 56.7% WNT: AR 5-yr PFS 100% HR 5-yr PFS 100%	[27]
SJMB12 Reduction	W1: M0, R0	1500 cGy CSI 5100 cGy PF boost	4 cycles VCR 8 mg/m^2^ CDDP 300 mg/m^2^ CPM 12 mg/m^2^	W1 stratum: 5-yr PFS 90.4%	[32]
HIT/SIOP PNET-4	M0, R0/R+	Randomized: STRT 2340 cGy CSI 5400 cGy PF HFRT 3600 cGy CSI 6000 cGy PF boost	Concurrent VCR 8 cycles VCR 48 mg/m^2^ CDDP 560 mg/m^2^ CCNU 600 mg/m^2^	5-yr PFS was 77% (STRT) vs. 78% (HFRT) WNT 5-yr PFS 91%	[33,34,35]
SIOP PNET-5	LR: M0, R0 WNT HR: M+, R+	LR 1800 cGy CSI 5400 cGy boost WNT HR: 2340 cGy CSI 5400 cGy boost	LR and WNT-HR <16 yrs 6 cycles (BABABA) VCR 18 mg/m^2^ CDDP 210 mg/m^2^ CCNU 225 mg/m^2^ CPM 6 mg/m^2^ WNT-HR^3^ 16 yrs 8 cycles (BABABABA) VCR 24 mg/m^2^ CDDP 280 mg/m^2^ CCNU 300 mg/m^2^ CPM 8 mg/m^2^	Active, not recruiting	[36]

AR, average risk; CBP, carboplatin; CCNU, lomustine; CDDP, cisplatin; CPM, cyclophosphamide; CSI, cranial spinal irradiation; ETOP, etoposide; HFRT, hyperfractionated radiation therapy; HR, high risk; IFRT, involved-field radiation therapy; LR, low risk; PF, posterior fossa; PFRT, posterior-fossa radiation therapy; PFS, progression-free survival; RT, radiation therapy; STRT, standard fractionated radiation therapy; VCR, vincristine.

**Table 2:** Historical trials using chemotherapy-only or radiation-delaying chemotherapy protocols for malignant brain tumors of early childhood.

Trial		N	5-y PFS	5 y OS	Reference
**M0R0**					
Baby POG	Medulloblastoma	13		69%	[40,41]
	sPNET	4	3 y PFS 100%		[41]
	HGG (all stages)	18	3 y PFS 43%	3 y OS 50%	[42]
CCG 9921	Medulloblastoma	38	41%	54%	[43]
	HGG (all stages) <2 y	32	35.3%	58.8%	[42]
POG 9233	sPNET	10	40%	50%	[44]
SFOP	Medulloblastoma	47	29%	73%	[45]
	HGG				
HIT-SKK 87	Medulloblastoma	17	10 y PFS 53%	10 y OS 59%	[46]
HIT-SKK 92	Medulloblastoma	17	82%	93%	[47]
	HGG				
CCG99703	Medulloblastoma	20	63.2%	68.4%	[48]
	sPNET	6	29%		
	Pineal PNET	4	29%		
	ATRT	5	37.5%		
Head Start I, II	Medulloblastoma Age <3 y	14	64%	86%	[49]
	HGG HS I Age <6 y	18	2 y PFS 11%	2 y OS 22%	[42]
**M0R1**					
Baby POG	sPNET	9	3 y PFS 11%		[41]
CCG 9921	Medulloblastoma	23	26%	40%	[43]
POG 9233	sPNET	19	15%	25%	[44]
SFOP	Medulloblastoma	17	6%	41%	[45]
HIT-SKK 87	Medulloblastoma	9	10 y PFS 56%	10 y OS 67%	
HIT-SKK 92	Medulloblastoma	14	50%	56%	[47]
CCG99703	Medulloblastoma	16	46.2%	61.5%	
	ATRT	3	37.5%		
Head Start I, II (Age < 3 y)	Medulloblastoma	7	29%	57%	[49]
**Metastatic (M+)**					
CCG 9921	Medulloblastoma	31	25%	31%	[43]
POG 9233	sPNET	9	10%	20%	[44]
SFOP	Medulloblastoma	15	13%	31%	[45]
HIT-SKK 87	Medulloblastoma	3	0%	0%	[46]
HIT-SKK 92	Medulloblastoma	12	33%	38%	[47]
CCG99703	Medulloblastoma	10	30%	50.5%	[48]
	sPNET	3			
	Pineal PNET	4			
	ATRT	0			
Head Start II (age <6 y)	Medulloblastoma	21	3 y PFS 49%	3 y OS 60%	[50]
**M/R status unspecified**					
Head Start I, II	HGG HS I Age <6 y	18	2 y PFS 11%	2 y OS 22%	[42]
	sPNET	43	39%	49%	[51]
ACNS0334	HR, MB, and sPNET	59	5 y EFS 68.2% for HR MB 5 y EFS 29.2% for sPNET	-	[52,53]
ACNS0333	ATRT	65	4 y EFS 37%	4 y OS 43%	[54]
HR MB-5	Medulloblastoma	28	3 y EFS 42.3%	3 y OS 71.3%	[55]

ATRT, atypical rhabdoid tumor; EFS, event-free survival; HGG, high-grade glioma; HR MB, high-risk medulloblastoma; PNET, supratentorial primitive neuroectodermal tumor; PFS, progression-free survival; OS, overall survival; sPNET, supratentorial primitive neuroectodermal tumor.

**Table 3:** Subtypes of pediatric high-grade glioma and molecular alterations (adapted from Chatwin, 2021 [113]).

Subtypes	Molecular Alterations
H3-mutant	H3K27M, H3G34R/V, ACVR1
IDH-mutant	IDH1/2
WT-A	BRAF V600E, NF1, or RTK fusions
WT-B	EGFR, CDK6, or MYCN amplifications
WT-C	PDGFRA and MET amplifications
Radiation-induced glioma/Therapy- induced HGG	TP53 mutations, PDGFRA/MET/BRAF amplifications, CDKN2A deletion
Infant-type	ALK, ROS1, NTRK, MET fusions
